# The Hsp90 Co-chaperones Sti1, Aha1, and P23 Regulate Adaptive Responses to Antifungal Azoles

**DOI:** 10.3389/fmicb.2016.01571

**Published:** 2016-10-05

**Authors:** Xiaokui Gu, Wei Xue, Yajing Yin, Hongwei Liu, Shaojie Li, Xianyun Sun

**Affiliations:** ^1^State Key Laboratory of Mycology, Institute of Microbiology, Chinese Academy of SciencesBeijing, China; ^2^College of Life Sciences, University of Chinese Academy of SciencesBeijing, China

**Keywords:** azole, drug resistance, Hsp90, *sti1*, *aha1*, *p23*, co-chaperone

## Abstract

Heat Shock Protein 90 (Hsp90) is essential for tumor progression in humans and drug resistance in fungi. However, the roles of its many co-chaperones in antifungal resistance are unknown. In this study, by susceptibility test of *Neurospora crassa* mutants lacking each of 18 Hsp90/Calcineurin system member genes (including 8 Hsp90 co-chaperone genes) to antifungal drugs and other stresses, we demonstrate that the Hsp90 co-chaperones Sti1 (Hop1 in yeast), Aha1, and P23 (Sba1 in yeast) were required for the basal resistance to antifungal azoles and heat stress. Deletion of any of them resulted in hypersensitivity to azoles and heat. Liquid chromatography–mass spectrometry (LC-MS) analysis showed that the toxic sterols eburicol and 14α-methyl-3,6-diol were significantly accumulated in the *sti1* and *p23* deletion mutants after ketoconazole treatment, which has been shown before to led to cell membrane stress. At the transcriptional level, Aha1, Sti1, and P23 positively regulate responses to ketoconazole stress by *erg11* and *erg6*, key genes in the ergosterol biosynthetic pathway. Aha1, Sti1, and P23 are highly conserved in fungi, and *sti1* and *p23* deletion also increased the susceptibility to azoles in *Fusarium verticillioides*. These results indicate that Hsp90-cochaperones Aha1, Sti1, and P23 are critical for the basal azole resistance and could be potential targets for developing new antifungal agents.

## Introduction

Invasive fungal diseases (IFDs), primarily caused by yeast-like fungi (e.g., *Candida albicans* and *Cryptococcus neoformans*) and filamentous fungi (e.g., *Aspergillus fumigatus*), are life-threatening infections with high morbidity and mortality in human, especially for immunocompromised patients, such as cancer patients undergoing chemotherapy and transplant recipients. Azoles, the most widely used antifungal agents, are still applied as the first-line therapy to treat patients suffering from IFDs because their side effects are lower than the “gold standard” polyenes such as Amphotericin B (AMB; Laniado-Laborín and Cabrales-Vargas, 2009). However, azole-resistant pathogenic fungi have frequently been isolated (Sheehan et al., 1999; Snelders et al., 2008; Howard et al., 2009). Surveillance studies indicate that azole-resistant *A. fumigatus* has spread throughout Europe, Asia, and Africa and can be detected in environmental and clinical settings (Howard et al., 2009; Bueid et al., 2010; Denning and Perlin, 2011). The evolution of antifungal resistance could render first-line azole treatment obsolete.

The direct target of azoles is the lanosterol 14α-demethylase ERG11/Cyp51, a key enzyme of ergosterol synthesis (Yoshida and Aoyama, 1987). Azoles bind to ERG11 and inhibit its activity, compromising cell membrane integrity by depleting ergosterol levels and/or causing an accumulation of the toxic intermediate 14α-methyl-3,6-diol (Kelly et al., 1995). Fungi make adaptive responses to azole stress by adjusting the transcriptional levels of a number of genes (Agarwal et al., 2003; da Silva Ferreira et al., 2006; Yu et al., 2007; Liu et al., 2010; Sun et al., 2014). Under antifungal stress, heat shock protein Hsp90, and its client proteins play important roles in establishing the resistant responses to azoles (Cowen and Lindquist, 2005; Cowen, 2013; Lamoth et al., 2013). Hsp90 governs many signal transduction pathways through chaperoning so-called “client proteins,” such as hormone receptors and protein kinases in eukaryotic cells (Young et al., 2001). Hsp90 stabilizes mutated oncogenic proteins, which are prone to misfolding, enabling malignant transformation in humans. Hsp90 assists protein folding and repairs misfolded proteins to maintain cellular proteostasis. In fungi, Hsp90 buffers the key regulators of cell signaling to cope with the stress of drug exposure (Cowen, 2009).

Intensive studies have been done to understand how Hsp90 mediates azole resistance in *Saccharomyces cerevisiae*, the yeast-like fungus *C. albicans*, and the filamentous fungus *A. fumigatus* (Cowen and Lindquist, 2005; Cowen et al., 2006; Cowen, 2009). Inhibition of Hsp90's ATPase activity by the natural products geldanamycin or radicicol reduces azole resistance in *S. cerevisiae* and *C. albicans* (Cowen and Lindquist, 2005; Zhang et al., 2013). Calcineurin is a key downstream client protein of Hsp90, which regulates numerous responses to environmental stimuli, including antifungal azoles. Calcineurin requires direct interaction with Hsp90 to maintain its stability and activation. Inhibiting the catalytic subunit (Cna1 or Cna2) by cyclosporine A or the regulatory subunit (Cnb1) by FKBP51 reduce azole resistance in *C. albicans* and *A. fumigatus* (Cruz et al., 2002; Uppuluri et al., 2008; Lamoth et al., 2013). Thus, the combination of antifungal drugs and Hsp90/Calcineurin inhibitors provides promising potential therapy for IFDs, which could also reduce the incidence of azole resistance (Cowen, 2009). Under fluconazole stress, *S. cerevisiae* Hsp90 promoted the rapid mutations in *erg3* that confers fluconazole resistance, suggesting Hsp90 is involved in the rapid evolution of drug resistance (Cowen, 2009).

The chaperone activity of Hsp90 requires successive binding to a series of co-chaperones in an ATP/ADP-dependent manner. The core co-chaperones include Cdc37, Sti1/Hop, peptidyl-prolyl cis-trans isomerases (PPIases; e.g., Cpr6/7, Cyp40, and FKBP51/52), Aha1, and P23/Sba1. These co-chaperones together with Hsp90 and Hsp70 comprise the regulation complex that governs stress responses induced by antifungal drugs, chemicals, and other environmental stresses. Deficiency in any co-chaperone protein compromises Hsp90 activity (Sullivan et al., 2002; Walton-Diaz et al., 2013). However, the roles of many co-chaperones in antifungal resistance are unknown. In this study, we investigated whether genetic deletion of these co-chaperones would affect Hsp90-mediated azole resistance in filamentous fungi. *Neurospora crassa* has transcriptional responses to ketoconazole (KTC) similar to that of pathogenic fungi (Zhang et al., 2012; Sun et al., 2013, 2014; Müller et al., 2015; Wang et al., 2015), and about 70% of the genes in *N. crassa* have knockout mutants, meaning *N. crassa* is an excellent model for identifying regulatory genes in drug resistance. By susceptibility test of *N. crassa* mutants lacking each of Hsp90 orchestrates member genes [*hsp80, hsp70-1, hsp70-2, hsp70-3, p23, aha1, sti1, cdc37, nup-17* (*PPIase*), *nup-5* (*PPIase B*), *nup-13* (*PPIase H*), and *fkr-5* (*PPIase FKBP-type*)], calcineurin encoding genes (*cna1* and *cnb1*), Hsp90/calcineurin-dependent stress responses genes (*crz1* and *hsf1*) and other heat shock protein family genes (*hsp88* and *hsp98*) to antifungal drug ketoconazole, we found that the Hsp90 co-chaperones Sti1, Aha1, and P23 participated in adaptive responses to azoles.

## Materials and methods

### Strains and cultural conditions

All *N. crassa* strains used in this study are listed in Table 1. *N. crassa* single-gene deletion mutants were purchased from Fungal Genetic Stock Center (FGSC, University of Kansas Medical Center, Lawrence, KS, USA). Double mutants Δ*p23*Δ*sti1* and Δ*p23*Δ*aha1* were generated by crossing FGSC#01792 (Δ*p23*) and FGSC#00714 (Δ*sti1*), and FGSC#01792 (Δ*p23*) and FGSC#04087 (Δ*aha1*), respectively, with the method described before (Zhang et al., 2012). Vogel's minimum medium, supplemented with 2% (w/v) sucrose for slants or 2% (w/v) glucose for plates, was used for culturing *N. crassa* strains. The slants were incubated at 28°C in the dark for 2 days and then in light for 5 days for conidiation. Antifungal compounds were added when needed. *Fusarium verticillioides* wild type M-3125 (mating type: MAT-1) and knockout mutants were cultured on potato dextrose agar at 28°C in the dark.

**Table 1:** *****N. crassa*** strains used in this study and their susceptibility to ketoconazole (KTC)**.

**Gene name**	**Annotation**	**Gene**	**Strain no**.	**Mating type**	Susceptibility to KTC^***^
WT			FGSC#4200	a	
*hsp80*	Heat shock protein 90	NCU04042	FGSC#11468	a, het^*^	++
			FGSC#11625	het	++
*p23/sba1*	Co-chaperone of HSP90	NCU01792	FGSC#11871	a	+++
			FGSC#11872	A	+++
*aha1*	Activator of HSP90 ATPase	NCU04087	FGSC#11561	A	+++
*sti1*	Stress inducible protein	NCU00714	FGSC#18705	A	+++
*hsf1*	Heat shock transcription factor	NCU08512	FGSC#14521	a, het	++
*cna1*	Calcineurin subunit A	NCU03804	FGSC#17929	a, het	+
			FGSC#11549	NA^**^	++
			FGSC#11550	NA	++
*cnb1*	Calcineurin subunit B	NCU03833	FGSC#12512	Het	+
			FGSC#12496	a, NA	+
			FGSC#15793	a, NA	+
*cdc37*	Cell division cycle 37	NCU00472	FGSC#16472	a, het	+
*hsp70-1*	Heat shock protein 70	NCU09602	FGSC#16015	a, het	−
*hsp70-2*	Heat shock protein 70	NCU02705	FGSC#13509	a, het	+
*hsp70-3*	Heat shock protein 70 cofactor	NCU01499	FGSC#14664	a	+
*hsp88*	HSP70 family, HSPA4-like	NCU05269	FGSC#11627	Het	−
*hsp98*	HSP110 family, HSP104-like	NCU00104	FGSC#11558	a	−
*nup-17* (*PPIase*)	Cyclophilin 40 family	NCU03853	FGSC#11560	a	−
*nup-5 (PPIase B)*	Cyclophilin 40 family	NCU01200	FGSC#12036	a	+
*nup-13 (PPIase H)*	Cyclophilin 40 family	NCU02614	FGSC#11922	a	+
*fkr-5 (PPIase FKBP-type)*	FKBP52 family	NCU02455	FGSC#12999	a	−
*crz1*	Calcineurin-responsive zinc finger	NCU07952	FGSC#11494	a, het	−
*Δp23Δsti1*	double mutation	NCU01792, NCU00714	This study		
*Δp23Δaha1*	double mutation	NCU01792, NCU04087	This study		

* *het, heterokaryon*;

** *NA, Not Available (in FGSC)*;

*** *Susceptibility to 2 mg/L ketoconazole was measured by colony diameters, the diameters of wild type and tested strains were marked as R and r, “−” represents r ≈ R, “+” represents 50%R < r < R, “++” represents 0 < r < 50%R, and “+++” represents r ≈ 0*.

### Susceptibility tests

Ketoconazole (KTC), fluconazole (FLU), itraconazole (ITC), amphotericin B (AMB), caspofungin (CSP), and geldanamycin (GA) were dissolved in DMSO. The final DMSO concentrations in media were below 0.25% (v/v; Sun et al., 2013). Menadione and H_2_O_2_ were diluted in distilled water. The agents were aseptically added to media before plates were made. The final concentrations of KTC, FLU, ITC, AMB, CSP, GA, menadione, and H_2_O_2_ in the media were 2, 25, 10, 0.05, 1, 1, 2 mg/L, and 2 mM, respectively. Two microliters of conidial suspension (2 × 10^6^ spores/ml) were inoculated on plates (Φ 90 mm, 12 mL medium each) with or without drugs and the plates were incubated at 28°C in the dark. Heat sensitivity tests were carried out at 28 and 42°C, respectively. Each test was duplicated and was repeated at least three times.

### Determination of minimum inhibitory concentration (MIC) of azoles

The MICs of KTC and FLU for each strain were determined in 96-well microtiter plates according to previous methods (Zhang et al., 2012). Briefly, 100 μl of 2 × KTC or FLU solution and 100 μl of conidial suspension media were added to each well. The final KTC concentrations were 0, 0.25, 0.5, 1, 2, 3, 4, 5, and 6 μg/ml; the final FLU concentrations were 0, 5, 10, 20, 30, 40, 50, and 60 μg/ml. The final conidial concentration was approximately 1 × 10^5^ conidia/ml. The plates were incubated at 28°C for 24 h. The minimum inhibitory concentration (MIC) values were determined as the lowest KTC concentration that inhibited growth observed by naked eye and dissecting microscope.

### Complementation of *sti1, aha1*, and *p23* mutants in *N. crassa*

All primers used in this study are listed in Table S1. To create a complementary *p23* plasmid, the full-length *p23* coding sequence (947 bp) with 1733 bp upstream and 935 bp downstream was amplified using the primers Nc-p23-com-1(ClaI) and Nc-p23-com-2(EcoRV), yielding a 3734 bp complementary fragment. The PCR product was inserted into the plasmid pCB1532 (Sweigard et al., 1997), which harbors a sulfonylurea resistant allele of *Magnaporthe grisea* ILV1 as a selective marker, at ClaI and EcoRV sites to create pCB1532-p23. For *sti1*, a 4174 bp fragment, consisting of 1567 bp upstream, 1868 bp coding sequence, and 723 bp downstream of *sti1*, was created by PCR using the primers Nc-sti1-com-1(XbaI) and Nc-sti1-com-2(BamHI). The fragment was then inserted into pCB1532 at BamHI and XbaI sites to create pCB1532-sti1. Similarly, a 3707 bp PCR fragment, including 1771 bp upstream, 1189 bp coding sequence, and 747 bp downstream of *aha1*, was amplified with the primers Nc-aha1-com-1 and Nc-aha1-com-2, and then ligated with linearized pCB1532, by EcoRV digestion, to generate pCB1532-aha1. The constructs pCB1532-p23, pCB1532-sti1 and pCB1532-aha1 were transformed into Δ*p23*, Δ*sti1*, and Δ*aha1* mutants, respectively, using the method described previously (Sun et al., 2012) Transformants were screened on Vogel's medium with 20 μg/ml chlorimuron ethyl and verified by PCR.

### RNA extraction and quantitative RT-PCR analysis

The culture conditions for RNA extraction were the same as previously described by Sun et al. (2012). Briefly, wild type and mutant conidia were separately inoculated into 20 mL liquid medium in a plate (Φ 90 mm) and incubated for 24 h at 28°C in the dark to form mycelia mats on the surface of the liquid medium. The mycelia mats were then cut into small pieces (Φ 10 mm), which were then torn into fragments (< 5 mm^2^) and transferred to 100 mL liquid media in 150 mL Erlenmeyer flasks. Cultures were incubated at 28°C with shaking at 180 rpm for 12 h. KTC was then added into the medium at a final concentration of 2.5 mg/L, as required. After 24 h incubation, mycelia were harvested and total RNA was extracted and reverse transcribed using FastQuant RT Kit (with gDNase) (Tiangen Biotech Co. Ltd., Beijing, China). The synthesized cDNA (2 μg in 20 μl) was diluted to 200 μl, and quantitative PCR was carried out on a CFX-96 Multicolor Real-Time PCR Detection System (Bio-Rad, Hercules, CA, USA) with SYBR-Green detection (SYBR Master Mix, TOYOBA Biotechnology Co., Ltd., Osaka, Japan); 0.8 μl each forward and reverse primers, 6 μl diluted cDNA, and 10 μl SYBR master mix were mixed in a 20 μl reaction, obtaining a 3 ng/μl final cDNA concentration. Each cDNA sample was analyzed in duplicate and repeated at least three times, and the average threshold cycle was calculated. Relative expression levels were calculated using the 2^−ΔΔCt^ method (Livak and Schmittgen, 2001). The results were normalized to the level of β-tubulin.

### Sterol extraction and analysis

Sterol extraction and liquid chromatography–mass spectrometry (LC-MS) analysis were performed as described by Sun et al. (2013). Briefly, 0.1 g dried mycelia powder with 25 μg fluconazole was extracted in 1.7 ml chloroform for 12 h with 10 min ultrasonic treatments before and after extraction. The extracts were dried and dissolved in 300 μl methanol under ultrasonication for 30 min. The extracts were subjected to LC-MS analysis as previously described (Sun et al., 2013) after filtered with 0.22 μm Millipore filters. The derived sterols were identified with reference molecular weight and fragmentation spectra for known standards. The amounts of sterols were normalized by relative peak area with fluconazole as reference.

### Analysis of co-chaperone homologs in fungi and human

The homologs of Hsp90 co-chaperones were identified in the National Center for Biotechnology Information (NCBI) by protein-protein BLAST (http://www.ncbi.nlm.nih.gov/BLAST) from the following species: *N*. *crassa* (taxid: 5141), *F. oxysporum* (taxid: 5507), *A. fumigatus* (taxid: 746128), *C. albicans* (taxid: 5476), *S. cerevisiae* (taxid: 4932), *C. neoformans* (taxid: 5207), and *Homo sapiens* (taxid: 9606). To reveal the sequence conservation and phylogenic relationship of homologs of Hsp90 and its three co-chaperones in fungi and human, the peptide sequences were aligned with DNAman software, and the results of multiple alignment were shown by phylogenetic trees generated with the Neighbor-Joining method (bootstrap = 1000) under support of MEGA 7.0 (Kumar et al., 2016).

### Knockout of *sti1* and *p23* homologs in *F. Verticillioides*

To knockout the *sti1* and *p23* homologs (*sti1*: FVEG_00423, the *E*-value, query coverage and identity with *N. crassa sti1* are 8e^−72^, 62 and 72%, respectively; *p23*: FVEG_11505, the *E*-value, query coverage and identity with *N. crassa p23* are 0, 100 and 76%, respectively) in *F. verticillioides*, the upstream and downstream flanking sequences were amplified and ligated to the hygromycin phosphotransferase gene by fusion PCR. Then, the deletion cassettes were transformed into *F. verticillioides* wild type M-3125, producing deletion mutants. Fungal transformation followed the protocol described by Sun et al. (2013). Transformants were checked using the primers Fv-sti1-check-1/2 and Fv-p23-check-1/2, respectively.

## Results

### Deletion of *sti1, aha1*, or *p23* causes azole hypersensitivity in *N. crassa*

**Figure 1:**
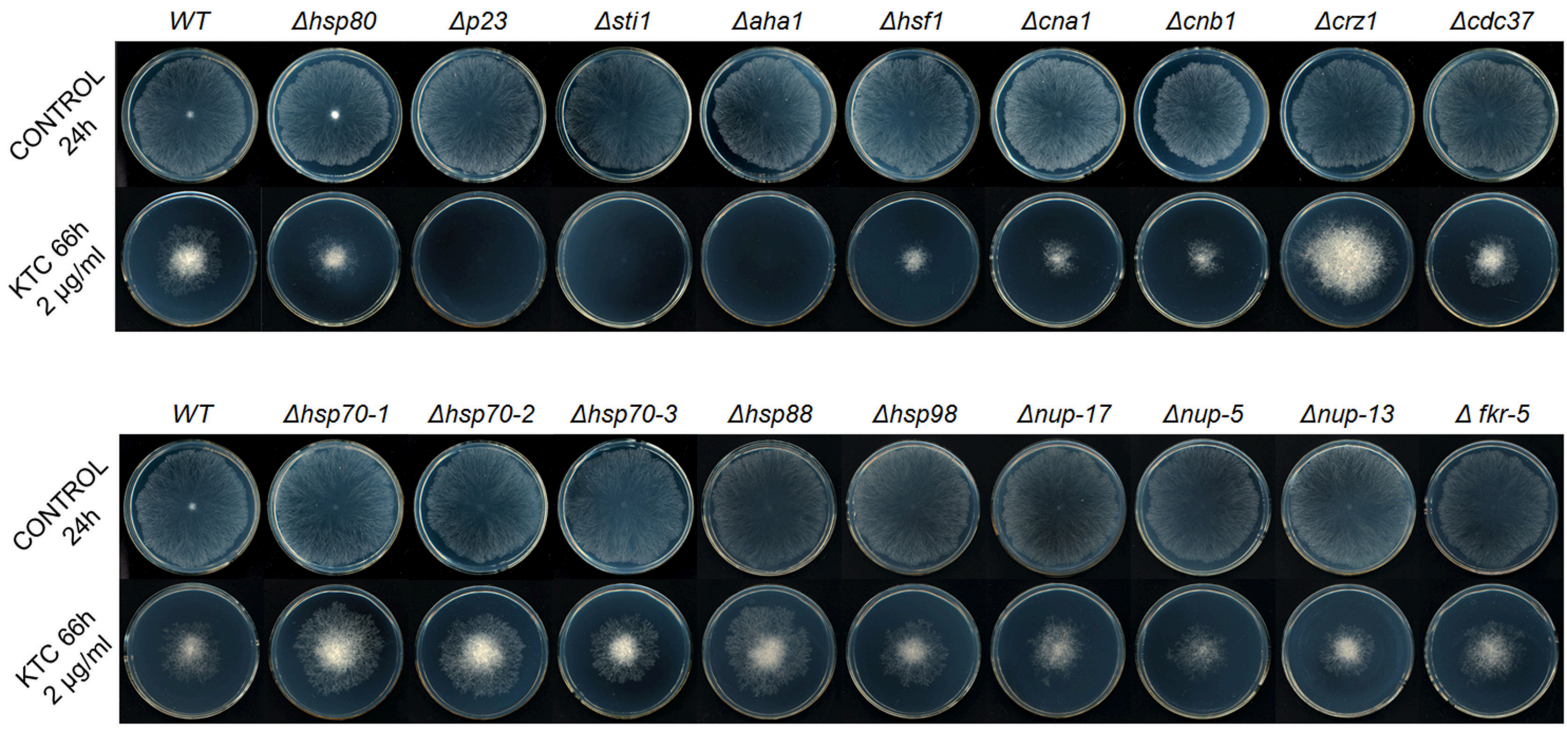
**Susceptibility tests of wild-type ***N. crassa*** and the knockout mutants of Hsp90 orchestrates member genes [***hsp80***, ***hsp70-1***, ***hsp70-2***, ***hsp70-3***, ***p23***, ***aha1***, ***sti1***, ***cdc37***, ***nup-17*** (***PPIase***), ***nup-5*** (***PPIase B***), ***nup-13*** (***PPIase H***), and ***fkr-5*** (***PPIase FKBP-type***)], calcineurin encoding genes (***cna1*** and ***cnb1***), Hsp90/calcineurin-dependent stress responses genes (***crz1*** and ***hsf1***) and other heat shock protein family genes (***hsp88*** and ***hsp98***) to antifungal drug ketoconazole (KTC)**. Two microliters of conidial suspension (2 × 10^6^ conidia/ml) were inoculated in the center of plates (Φ 90 mm) with or without antifungal drug, then incubated at 28°C for the indicated times. Each test was duplicated and the experiment was independently repeated at least three times.

**Figure 2:**
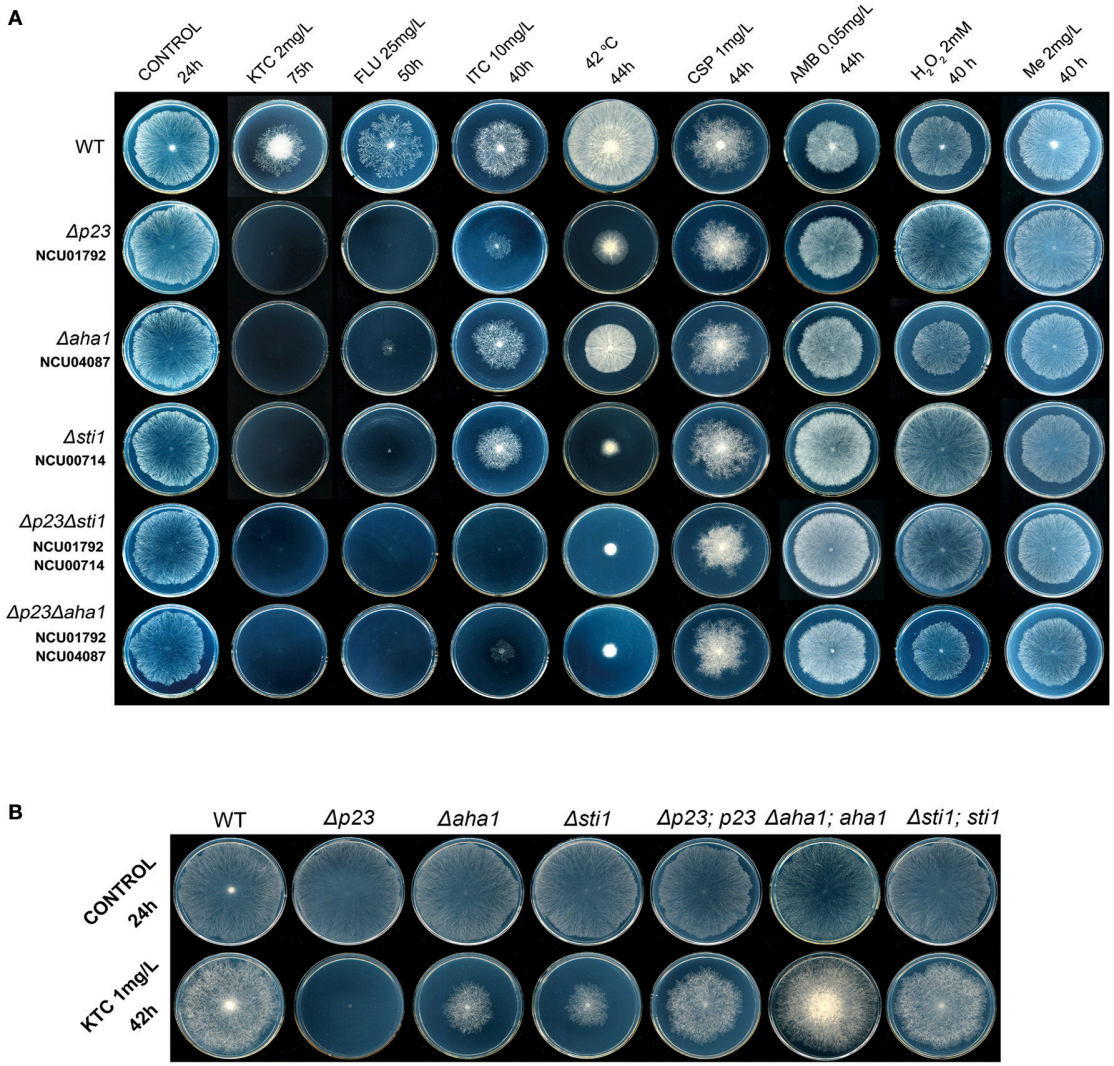
**Susceptibility tests of ***N. crassa*** to antifungal drugs, oxidants and heat stress. (A)** Susceptibility tests of wild-type *N. crassa*, the knockout mutants of *p23, aha1, sti1* (Δ*p23*, Δ*aha1*, and Δ*sti1*) and double deletion mutants (Δ*p23*Δ*aha1* and Δ*p23*Δ*sti1*) to antifungal drugs, oxidants and heat stress; **(B)** Susceptibility tests of the *N. crassa* knockout mutants Δ*p23*, Δ*aha1*, and Δ*sti1* and their complemented strains Δ*p23;p23*, Δ*aha1;aha1*, and Δ*sti1;sti1* to Ketoconazole. Two microliters of conidial suspension (2 × 10^6^ conidia/ml) were inoculated in the center of plates (Φ 90 mm) with or without antifungal drugs or oxidants, then incubated at 28 or 42°C (heat tests) for the indicated times. Each test was duplicated and the experiment was independently repeated at least three times. KTC, ketoconazole; FLU, fluconazole; ITC, itraconazole; CSP, caspofungin; AMB, amphotericin B; Me, menadione.

To investigate the roles of the Hsp90 co-chaperones in azole-induced stress responses, we tested the susceptibility of 24 single-gene deletion mutants or heterokaryons to 2 mg/L ketoconazole (KTC; Figure 1, Table 1). These genes were presumed to be involved in the Hsp90 cycle and calcineurin pathway. Without KTC, growth rates of all tested mutants were similar to that of wild type. On plates with 2 mg/L KTC, three mutants displayed much severer growth inhibition than the rest of mutants and wild type. The corresponding genes of these three mutants encode Hsp90 co-chaperones P23, Aha1, and Sti1, respectively (Figures 1, 2A, Table 2). Heterokaryons deficient for *hsp90* (the gene name is *hsp80*), or *cna1* (calcineurin catalytic subunit) or *cnb1* (calcineurin regulatory subunit), showed increased susceptibility to KTC. Deletion of *hsf1*, the gene coding a heat shock transcriptional factor, also increased susceptibility to KTC (Figure 1, Table 2). However, the mutants lacking either PPIases and heterokaryons deficient for Hsp70 showed wild-type KTC susceptibility (Figure 1, Table 1).

**Table 2:** **Relative inhibition rates of HSP90- related mutants by azoles, heat and H_**2**_O_**2**_**.

**Strain**	**Relative inhibition rate**				
	**KTC 2 μg/ml (%)**	**FLU 25 μg/ml (%)**	**ITA 10 μg/ml (%)**	**HEAT 42°C (%)**	**H_2_O_2_ 2.5 mm (%)**
WT	66.5 ± 0.3	65.2 ± 0.8	50.0 ± 0.3	44.5 ± 0.2	51.3 ± 0.4
*Δhsp80*	76.3 ± 0.6	74.2 ± 0.7^**^	51.9 ± 0.4^**^	50.0 ± 0.4	100 ± 0.0^**^
*Δp23*	100 ± 0.0^**^	100 ± 0.0^**^	78.3 ± 0.3^**^	70.9 ± 0.5^**^	28.7 ± 0.4^*^
*Δaha1*	100 ± 0.0^**^	98.5 ± 0.3^**^	57.4 ± 0.3	55.5 ± 0.3^*^	57.4 ± 0.5
*Δsti1*	100 ± 0.0^**^	100 ± 0.0^**^	62.3 ± 0.1^**^	75.0 ± 0.8^**^	26.3 ± 0.7^*^
*Δhsf1*	85.0 ± 1.6^*^	80.9 ± 0.8^**^	53.7 ± 0.2	56.7 ± 0.4^*^	54.4 ± 0.2
*Δcna1*	92.7 ± 0.3^*^	81.9 ± 0.6^**^	49.7 ± 0.3	49.2 ± 0.1	58.2 ± 1.6
*Δcnb1*	71.7 ± 0.7	65.0 ± 0.5	45.0 ± 0.3	45.7 ± 0.1	100 ± 0.0^**^
*Δp23Δsti1*	100 ± 0.0^**^	100 ± 0.0^**^	100 ± 0.0^**^	88.1 ± 0.1^**^	28.1 ± 0.8^*^
*Δp23Δaha1*	100 ± 0.0^**^	100 ± 0.0^**^	80.0 ± 0.1^**^	81.7 ± 0.2^**^	55.5 ± 0.4

Tested strains are the wild type (WT), the p23, aha1 and sti1 knockout strains (Δp23, Δaha1, and Δsti1), the hsp80, hsf1, cna1, and cnb1 heterokaryons (Δhsp80, Δhsf1, Δcna1, and Δcnb1) as well as double mutants Δp23Δsti1 and Δp23Δaha1. The means of the relative inhibition rates for each fungicide were calculated by the equation: (the growth rate on plates without fungicide – the growth rate on plates with the fungicide)/growth rate on plates without fungicide. Differences between mutants and the WT were statistically analyzed by the LSD and Dunnett T-tests (homoscedastic) or Tamhane's T2 and Dunnett C-tests (heteroscedastic). Values that are extremely significantly different (P < 0.01) are marked with ^**^ and values that are significantly different (0.01 < P < 0.05) are marked with ^*^.

Based on the above results, Hsp90 co-chaperones P23, Aha1, and Sti1 were chosen for further study. Complementation of Δ*sti1*, Δ*aha1*, and Δ*p23* recovered wild-type susceptibility to KTC (Figure 2B). The susceptibilities of mutants (Δ*p23*, Δ*aha1*, and Δ*sti1*) to fluconazole and itraconazole were also tested. The results obtained from fluconazole treatment corresponded to the results in KTC. On plates with 25 mg/L fluconazole, the growth of wild type was slower than that on the plates without drug; the growth of Δ*p23* was completed arrested; Δ*aha1* and Δ*sti1* formed only tiny colonies after 50 h incubation (Figure 2A). On plates with 10 mg/L itraconazole, the growth rates of these mutants and wild type were not dramatically different (Figure 2A).

### Each Hsp90 co-chaperone has independent contribution under azole stress

To further understand the relationship among these three co-chaperones under azole stress, double mutants Δ*p23*Δ*sti1* and Δ*p23*Δ*aha1* were generated. Interestingly, these double mutants displayed greater growth inhibition than the respective single gene deletion mutants on plates with itraconazole. On the plates with 10 mg/L itraconazole, the growth of Δ*p23*Δ*sti1* was completely arrested and Δ*p23*Δ*aha1* could only form tiny colonies, which were significantly smaller than those of Δ*p23* or Δ*aha1* (Figure 2A, Table 2). Since the susceptibility difference to KTC and fluconazole between each of double mutants and its respective single gene deletion mutants was difficult to show based on colony growth, MIC analysis was further conducted. Results showed that the double mutants Δ*p23*Δ*sti1* and Δ*p23*Δ*aha1* had MIC values to fluconazole significantly lower than their respective single-mutation lines (Table 3). For KTC, MIC values of these double mutants and their respective single gene mutant lines were not significantly different (Table 3). Since the complete suppression of single gene mutants' growth requires very low concentration of KTC (0.7–1.7 μg/ml), accurate measurement of differences in KTC susceptibility between each double mutant and the single gene mutant lines is difficult. Nevertheless, based on the results in itraconazole and fluconazole, it could be concluded that each of these Hsp90 co-chaperones has its independent role in the basal azole resistance.

**Table 3:** **Minimum inhibitory concentration (MIC) of ketoconazole and fluconazole for wild type and mutants (μg/ml)**.

	**WT**	***Δp*23**	***Δaha*1**	***Δsti*1**	***Δp*23*Δsti*1**	***Δp*23*Δaha*1**
KTC	3.0 ± 0.3	0.7 ± 0.3^**^	1.7 ± 0.3^*^	1.0 ± 0.0^**^	0.2 ± 0.1^**^	0.7 ± 0.3^**^
FLU	27 ± 5	10 ± 0^*^	20 ± 0^*^	17 ± 3^*^	5 ± 0^**^	7 ± 3^**^

Differences between the mutants and the wild type (WT) were statistically analyzed by the LSD and Dunnett T-tests (homoscedastic). Values that are significantly different (P < 0.01) are marked with ^**^ and values that are different (0.01 < P < 0.05) are marked with ^*^.

### The roles of p23, aha1, and sti1 in the basal azole resistance link to Hsp90

**Figure 3:**
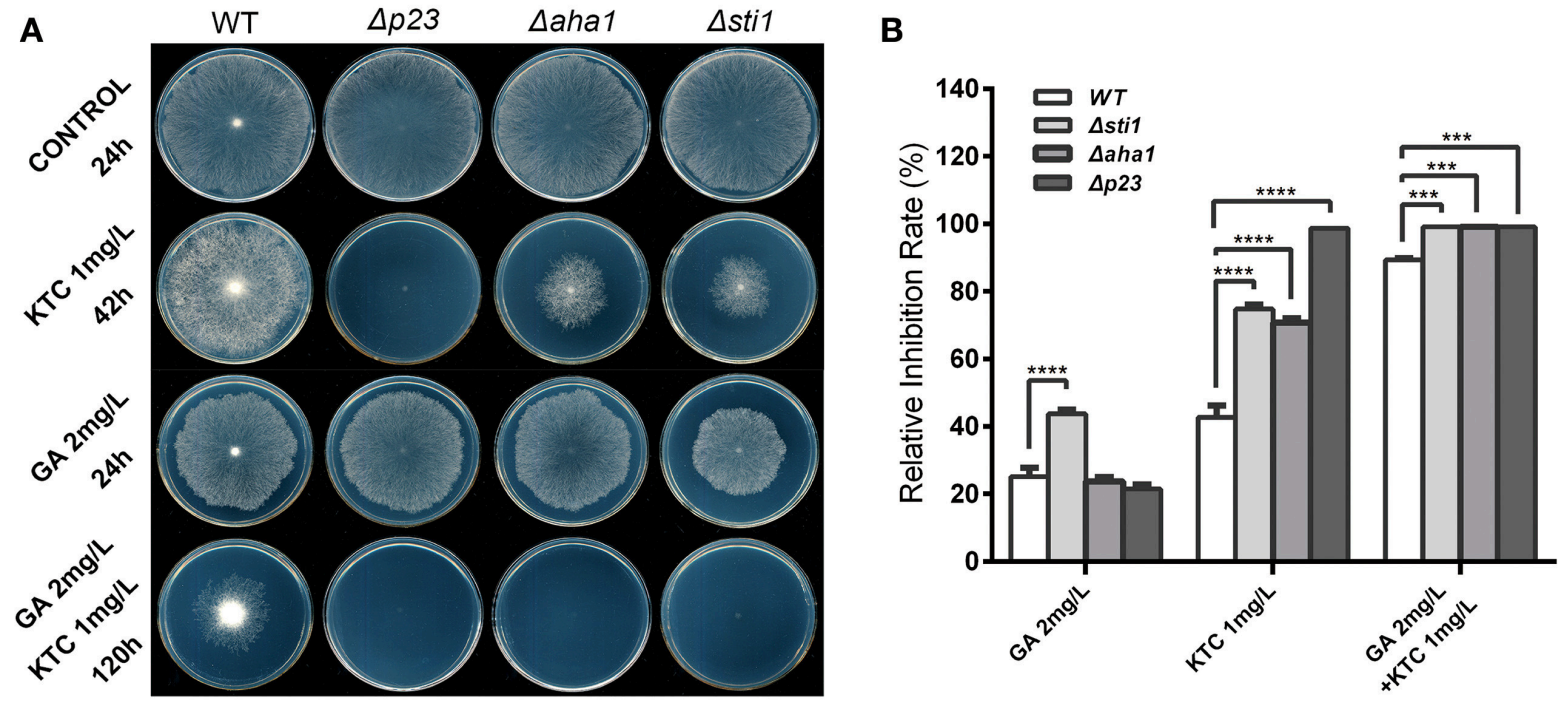
**Susceptibility tests of ***N. crassa*** to ketoconazole, the Hsp90 inhibitor geldanamycin and the two drugs combined. (A)** Susceptibility tests with geldanamycin (GA, 2 mg/L), ketoconazole (KTC, 1 mg/L) or the two drugs combined. Two microliters of conidial suspension (2 × 10^6^ conidia/ml) were inoculated in the center of plates (Φ 90 mm) with or without antifungal drugs or oxidants, and then incubated at 28 or 42°C (heat tests) for the indicated time. **(B)** Relative growth inhibition rates were calculated based on colony diameters at 24 h after drug treatment. Values from three replicates were used for a statistical analysis. Means of the inhibition rates are shown, and standard deviations are marked with error bars. Differences between the mutants and the WT were statistically analyzed by the analysis of variance. Values with *P* < 0.0001, 0.0001 < *P* < 0.001, 0.001 < *P* < 0.01, and 0.01 < *P* < 0.05 are marked with ^****^, ^***^, ^**^ and ^*^, respectively.

To further reveal the relationship between Hsp90 and its co-chaperones, the sensitivity of *N. crassa* strains to the Hsp90 inhibitor geldanamycin (GA) was tested. As shown in Figure 3, 2 mg/L GA affected *N. crassa* growth, but its growth effects in Δ*p23* and Δ*aha1* were similar to that of wild type. For Δ*sti1*, although its growth was only partially repressed, its growth was significantly slower than wild type. Thus, the simultaneous disruption of Hsp90 and its co-chaperones did not dramatically affect the hyphal growth on plates without antifungal azoles. Addition of 1 mg/L KTC slightly affected the growth of wild type but completely suppressed the growth of Δ*p23*. Although growing slower than wild type, Δ*aha1* and Δ*sti1* could form colonies on plates with 1 mg/L KTC after 42 h. On plates with both 2 mg/L GA and 1 mg/L KTC, the growth of all mutants was arrested. None of them formed visible colony even after 120 h incubation. Wild type could form colonies, but its growth was significantly slower than that on plates with 2 mg/L GA or 1 mg/L KTC alone. The above results suggest that Hsp90 and its co-chaperones P23, Aha1, and Sti1 play more important roles under azole stress than under normal conditions.

### Deletion of *sti1, aha1*, or *p23* did not increase susceptibility to amphotericin B and caspofungin

In addition to azoles, polyenes, and echinocandins are also widely used for treating fungal infections. To test whether Sti1, Aha1, and P23 are also required for the basal resistance to polyenes and echinocandins, susceptibilities of their mutants to amphotericin B and caspofungin were tested. As shown in Figure 2A, compared with wild type, neither single gene deletion mutants, including Δ*sti1*, Δ*aha1, and* Δ*p23*, nor double mutants, including Δ*p23*Δ*sti1* and Δ*p23*Δ*aha1*, displayed increased susceptibility to amphotericin B or caspofungin. These results indicate that Sti1, Aha1, and P23 are not generally required for the basal resistance to a wide range of antifungal drugs.

### Deletion of *sti1, aha1*, or *p23* causes hypersensitivity to heat in *N. crassa*

Hsp90 prevents ROS generation and stimulates anti-oxidative defenses under thermal stress conditions in the fungal species *Paracoccidioides brasiliensis* (Matos et al., 2013). We tested the roles of Hsp90 and its related genes under heat and oxidative stress. As shown in Figure 1, the growth rates of Δ*hsp80* and all tested mutants related to Hsp90 grew as normal as wild type at 28°C. At 42°C, Δ*p23*, Δ*aha1*, and Δ*sti1*, grew significantly slower than wild type (Figure 2A, Table 2). At 42°C, the growth of wild type was inhibited by 44.5% relative to its growth at 28°C, while the growth of Δ*sti1*, Δ*aha1*, and Δ*p23* was inhibited by 75, 55.5, and 70.9%, respectively, compared to their growth at 28°C. These results indicate that these Hsp90 co-chaperones also participate in stress responses induced by heat.

### Sti1 and p23 negatively regulate H_2_O_2_ resistance

H_2_O_2_ and menadione were used to test the roles of Hsp90 co-chaperones under oxidative stress. On plates with 2.5 mM H_2_O_2_, the growth of wild type was inhibited by 51.3%, relative to its growth on control plates (Table 2), while the growth of Δ*hsp80* and Δ*cnb1* were completely arrested (Table 2), which further demonstrated that Hsp90/calcineurin system had critical function under oxidative stress.In contrast, Δ*sti1* and Δ*p23* were more resistant than wild type. On plates with 2.5 mM H_2_O_2_, the growth of Δ*sti1* and Δ*p23* were inhibited by only 26.3 and 28.7%, respectively, which were significantly lower than that of wild type (by 51.3%; Table 2). Δ*aha1* displayed wild-type sensitivity to H_2_O_2._ On plates with 2 mg/L menadione, the growth of Δ*p23*, Δ*aha1*, and Δ*sti1* were comparable to wild type. Thus, these Hsp90 co-chaperones do not positively contribute to the basal resistance to oxidative stress as they do to azole stress. In contrast, Sti1 and P23 negatively regulate H_2_O_2_ resistance.

### Sti1, aha1, and p23 modulate the Hsp90 cycle and ergosterol biosynthesis at the transcriptional level

**Figure 4:**
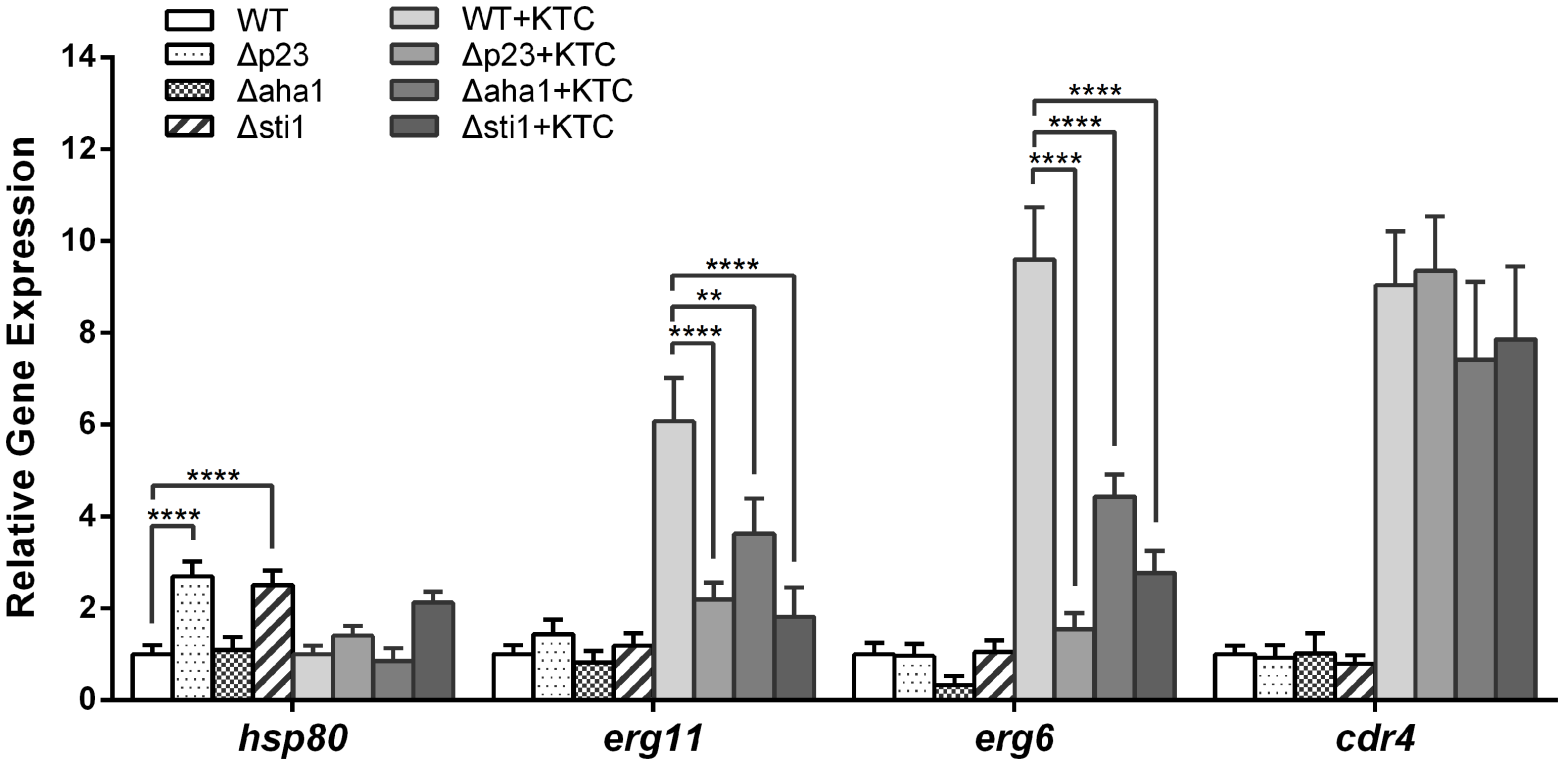
**Differential expressions of genes in the Δ***sti1***, Δ***aha1***, and Δ***p23*** strains relative to the wild type (WT) strain was determined by quantitative RT-PCR**. Strains were grown in liquid Vogel's medium at 28°C with shaking at 180 rpm for 12 h. Then ketoconazole (KTC) was added to the medium to reach 2.5 mg/L. After 24 h incubation, the following genes were analyzed by quantitative RT-PCR: *hsp80, erg11, erg6*, and *cdr4*. Values shown are the means of three independent replicates. Standard deviations are indicated by error bars, and differences between the mutants and WT were statistically analyzed by analysis of variance. Values with *P* < 0.0001, 0.0001 < *P* < 0.001, 0.001 < *P* < 0.01, and 0.01 < *P* < 0.05 are marked with ^****^, ^***^, ^**^ and ^*^, respectively.

Hsp90 governs the stress response network in fungi, and its co-chaperones are essential for the Hsp90 cycle (Walton-Diaz et al., 2013). To clarify how the co-chaperones affect the Hsp90 cycle at transcriptional level in *N. crassa*, we analyzed the expression of NCU04142 (*hsp80*, the Hsp90 coding gene), NCU00714 (*sti1*), NCU04087 (*aha1*), NCU01792 (*p23*), and NCU08512 (*hsf1*) using quantitative RT-PCR. In wild type *N. crassa*, KTC treatment did not affect the transcription of these genes (Data not shown), which is consistent with previous RNA-seq data (Sun et al., 2014). However, *hsp80* mRNA levels were more than 2-fold higher (2.7- and 2.3-fold, respectively) in both non-KTC and KTC treatments in Δ*sti1* than those of wild type; deletion of *p23* also caused elevated transcription of *hsp80* in non-KTC treatment (Figure 4). The increase of *hsp80* expression may be a compensatory effect of the loss of the co-chaperones, due to the Hsp90 auto-regulatory loop control (Leach et al., 2012).

Azoles target ERG11, directly blocking 14α-demethylation of lanosterol, resulting in sterol biosynthesis through the eburicol bypass and an accumulation of the toxic intermediate 14α-methyl-3,6-diol, which can damage membrane integrity (Kelly et al., 1995). The efflux pump CDR4 has been shown to be the major contributor to azole resistance among four Pdr5p-like ABC transporters in *N. crassa* (Zhang et al., 2012). We analyzed the expression of the key genes in ergosterol synthesis, including NCU02624 (*erg11*) and NCU03006 (*erg6*), as well as the efflux pump gene NCU05591 (*cdr4*), in their mutants and wild type. Quantitative RT-PCR revealed that in wild type, *erg11, erg6*, and *cdr4* were up-regulated by 6.0-, 7.6-, and 9.0-fold after 24 h of KTC (2.5 mg/L) treatment, respectively. These results are consistent with previous RNA-seq data (Sun et al., 2013). In contrast, the level of *erg11* in Δ*sti1*, Δ*aha1*, and Δ*p23* mutants was increased only 1.5-, 4.5-, and 1.7-fold compared with non-KTC treated wild type, respectively, and the levels of *erg6* in Δ*sti1* and Δ*p23* mutants were increased only 2.7- and 1.6-fold compared with non-KTC treated wild type, respectively. In Δ*aha1, erg6* levels were reduced by 0.33-fold without KTC treatment and increased 4.4-fold with KTC treatment compared with non-KTC treated wild type. The mRNA levels of *cdr4* in three mutant strains were similar to that of wild type in the medium without KTC. After KTC treatment, *cdr4* transcripts in mutants and wild type were elevated to a similar level (Figure 3). These data suggest that Sti1, Aha1, and P23 are required for the adaptive responses by genes involved in ergosterol synthesis under azole stress.

### Deletion of *sti1, aha1*, and *p23* causes excessive accumulation of toxic sterol 14α-methyl-3,6-diol

**Figure 5:**
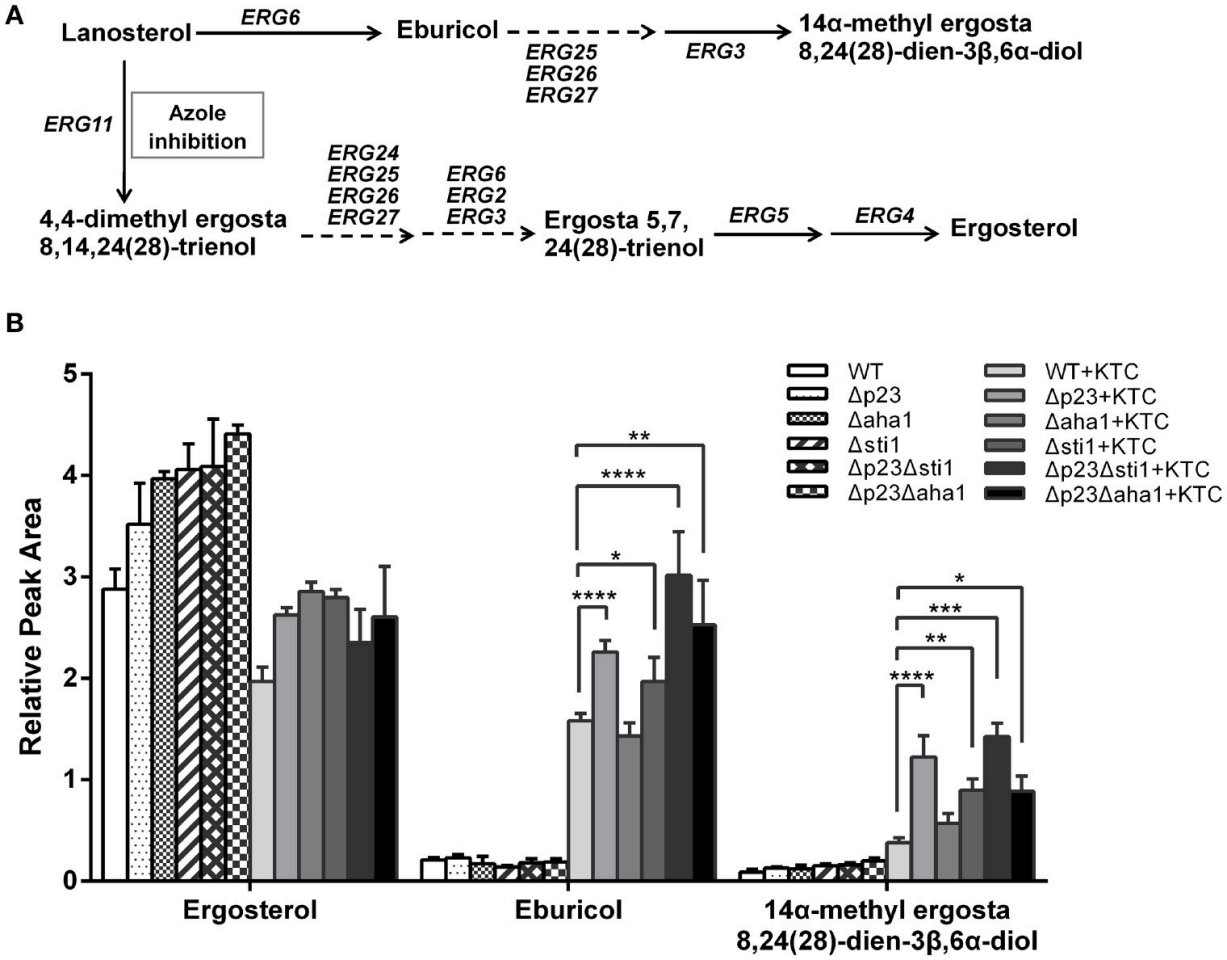
**Schematic representations of the ergosterol biosynthetic pathway (A) and quantification of sterol accumulations (B) in wild-type ***N. crassa*** and the knockout mutants**. Strains were grown in liquid Vogel's medium at 28°C with shaking at 180 rpm for 12 h. Then ketoconazole (KTC) was added to the medium to reach 2.5 mg/L. After 24 h incubation, ergosterol, eburicol, and 14α-methyl-3,6-diol were analyzed by LC-MS with fluconazole as a standard reference. Values shown are the means of three independent replicates. Standard deviations are indicated by error bars. Differences between the mutants and the WT were statistically analyzed by analysis of variance. Values with *P* < 0.0001, 0.0001 < *P* < 0.001, 0.001 < *P* < 0.01, and 0.01 < *P* < 0.05 are marked with ^****^, ^***^, ^**^, and ^*^, respectively.

Previous studies have shown that the mechanism of action of antifungal azoles includes decreasing ergosterol levels and/or the accumulation of 14α-methyl-3,6-diol (Kelly et al., 1995; Sun et al., 2014). Therefore, we tested the sterol composition in the mutants and wild type after 24 h culture with or without KTC (2.5 mg/L) using LC-MS with fluconazole as a reference to normalize the total amount of sterols. In wild type, ergosterol was the primary sterol (2.9, relative amount) and small amount of eburicol (0.2) was detected in untreated samples; when KTC was added, ergosterol levels were slightly reduced (2.0) but eburicol significantly increased (1.6) and 14α-methyl-3,6-diol was detected (0.4). The untreated mutants accumulated ergosterol as the primary sterol, similar to wild type. Following KTC treatment, ergosterol was reduced in Δ*p23*, Δ*aha1*, and Δ*sti1* (2.6/3.5, 2.9/4.0, and 2.8/4.1, respectively, KTC treatment/untreated); ergosterol was reduced more in the double mutants Δ*p23*Δ*sti1* and Δ*p23*Δ*aha1* (2.3/4.1 and 2.6/4.4, respectively). Meanwhile, eburicol and 14α-methyl-3,6-diol were sharply increased in these mutants following KTC treatment: Δ*p23*: 2.3/1.2; Δ*aha1*: 1.4/0.6; Δ*sti1*: 2.0/0.9; Δ*p23*Δ*sti1*: 3.0/1.4; Δ*p23*Δ*aha1*: 2.5/0.9 (eburicol/14α-methyl-3,6-diol; Figure 5). Thus, the excessive accumulation of toxic 14α-methyl-3,6-diol might be an important cause to the reduced azole resistance in these mutants.

### Hsp90 co-chaperones are highly conserved in filamentous fungi

Multiple sequence alignment revealed that the homologs of Hsp90 from seven fungal species, including *N. crassa, S. cerevisiae, C. albicans, F. verticillioides, F. oxysporum, A. fumigatus*, and *C. neoformans*, and *H. sapiens*, are highly conserved in amino acid sequences (Figure S1). However, the phylogenetic analysis shows that Hsp90s from fungi are distantly related to Hsp90s from *H. sapiens*: fungal Hsp90s were clustered to one big clade while human Hsp90s were clustered to another big clade (Figure S1). The similar results were obtained when homologs of Sti1, Aha1, and P23 were analyzed (Figures S2–S4).

### Knocking out the *p23* and *sti1* homologs in *F. verticillioides* increases sensitivity to KTC

**Figure 6:**
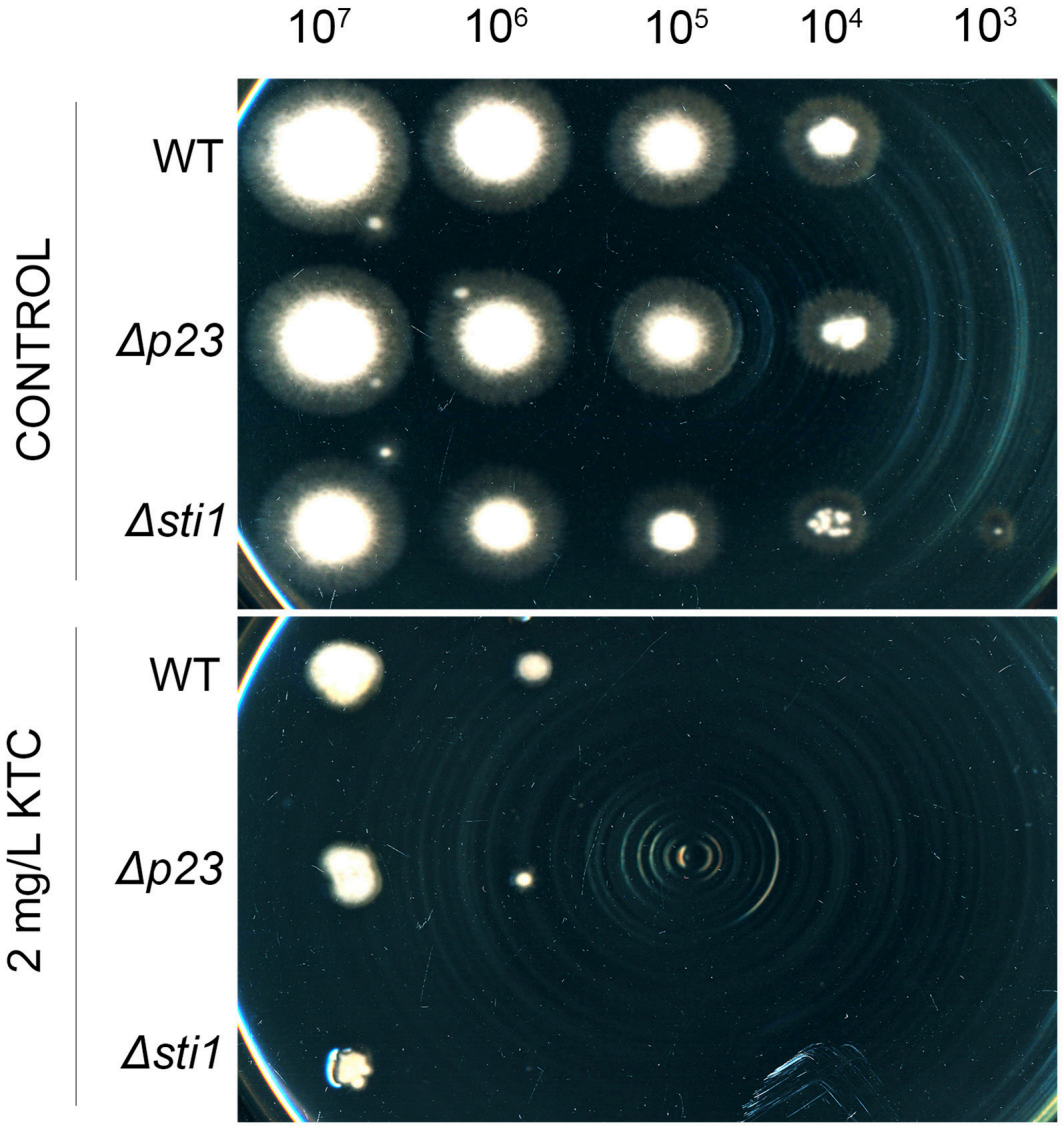
**The ***Fusarium verticillioides sti1*** homolog FVEG_00423 and ***p23*** homolog FVEG_11505 knockout mutants were hypersensitive to ketoconazole (KTC)**. Two microliters of conidial suspensions of different concentrations (1 × 10^7^, 1 × 10^6^, 1 × 10^5^, 1 × 10^4^, and 1 × 10^3^ conidia/ml) were inoculated onto the plates (Φ 150 mm) of potato dextrose agar medium with or without 2 mg/L KTC, and incubated at 28°C for 72 h. Each test had three replicates and the experiment was independently repeated twice.

To test the functional conservation of Sti1 and P23 among different fungi under azole stress, the gene deletion mutants for the *sti1* (FVEG_00423) homolog and the *p23* (FVEG_11505) homolog in plant pathogen *F. verticillioides*, were respectively created. As shown in Figure 6, on the medium without KTC, the growth rates of *sti1* and *p23* deletion mutants were similar to that of wild type. When grown on the medium with 2 mg/L KTC, both *sti1* and *p23* deletion mutants displayed increased sensitivity compared with wild type, indicating that Sti1 and P23 homologs also contributes to the basal azole resistance in *F. verticillioides* and their roles in azole adaptation should be conserved among fungi.

## Discussion

Upon fluconazole stress, Hsp90 promoted rapid accumulation of mutations that elevated resistance to fluconazole in *S. cerevisiae* and *C. albicans* (Cowen, 2009). Based on these lines of evidence, Hsp90 was believed to enable the rapid evolution of azole resistance (Cowen, 2009). Hsp90 might also mediate azole resistance by its regulatory role in protein phosphatase calcineurin. Hsp90 binds the catalytic subunit of calcineurin (Imai and Yahara, 2000). Calcineurin plays a pivotal role in the basal resistance to antifungal azoles in *C. albicans, C. neoformans*, and *A. fumigatus* (Juvvadi et al., 2016). In *S. cerevisiae* and *C. albicans*, calcineurin could dephosphorylate transcription factor Crz1 to activate transcriptional responses to stresses (Cyert, 2003). Crz1 is required for the basal resistance to fluconazole in *C. albicans* (Onyewu et al., 2004). Functioning as a chaperone, Hsp90 requires successive binding to a series of co-chaperones in an ATP/ADP-dependent manner. Recent studies showed that the Hsp90 co-chaperone Sgt1 is required for the basal resistance to azoles and echinocandins in *C. albicans*, and StiA (Hop) contributes caspofungin tolerance and resistance in *A. fumigatus* (Shapiro et al., 2012; Lamoth et al., 2015). However, the roles of many other co-chaperones in responses and resistance to antifungals are unknown. By analyzing effects of gene disruption of 18 components of Hsp90 system on ketoconazole susceptibility, we showed that disruption of Hsp90, calcineurin subunit A (Cna1), calcineurin subunit B (Cnb1), and the transcription factor Hsf1 which regulates the expression of Hsp90, compromised the basal resistance to ketoconazole in *N. crassa*. These results indicate that the Hsp90-dependent resistance to azole stress in the model fungal species *N. crassa* is similar to that in pathogenic fungi *C. albicans* and *A. fumigatus*. Among eight analyzed Hsp90 co-chaperones, the mutants defective in Sti1, Aha1, and P23 displayed severer defects than the rest co-chaperone mutants under ketoconazole stress, suggesting these three Hsp90 co-chaperones play important roles under azole stress.

The core co-chaperones Sti1, Aha1, and P23 are essential for the Hsp90 cycle. In the early stage of the Hsp90 cycle, Sti1 functions as a transporter of client proteins from Hsp70 to Hsp90, as well as an Hsp90 ATPase inhibitor to stabilize the Hsp90-Hsp70-Sti1-client complex (Richter et al., 2003). In the late stage of the Hsp90 cycle, Aha1 promotes the hydrolysis of ATP to supply energy and activate the conformational changes of the Hsp90-client protein complex (Retzlaff et al., 2010). P23 is the rate limiting component of the Hsp90 cycle, which stabilizes the Hsp90-P23-client complex and inhibits the intrinsic Hsp90 ATPase activity to prolong the interaction until the protein folding process is completed (Morishima et al., 2003; Ali et al., 2006). We demonstrate that the roles of Sti1, Aha1, and P23 under azole stress link to Hsp90 by showing that inhibiting Hsp90 by geldanamycin further increased the susceptibility of their deletion mutants to ketoconazole. Simultaneous deletion of *p23* and *sti1* or *aha1* caused severer growth defects under itraconazole or fluconazole stress than the single gene deletion mutants. Thus, each of these co-chaperones has its own independent contribution to Hsp90-dependent azole resistance.

In response to azole stress, fungi up-regulate transcriptional levels of a number of genes (Agarwal et al., 2003; da Silva Ferreira et al., 2006; Liu et al., 2010; Sun et al., 2014), among which transcriptional up-regulation of genes involved in ergosterol biosynthesis and azole efflux pumps has been demonstrated to be able to elevate resistance to azoles (White, 1997; Bueid et al., 2010; Denning and Perlin, 2011; Cools et al., 2013). Our results indicate that Sti1, Aha1, and P23 are not important for the transcriptional response to ketoconazole by the key azole efflux CDR4, the ortholog of yeast Pdr5p (Zhang et al., 2012). However, Sti1, Aha1, and P23 are required for the normal responses to ketoconazole by *erg11* (the target gene of azoles) and *erg6* (another gene essential for ergosterol biosynthesis). Deletion of *p23* and *sti1* had greater effects on the transcriptional responses by *erg11* than deletion of *aha1*. This might provide an explanation to why the *sti1* mutant and the *p23* mutant are more susceptible to fluconazole and itraconazole than the *aha1* mutant. In consistence with results at RNA level, sterol analysis showed that the *sti1* mutant and the *p23* mutant accumulate more toxic intermediates than wild type and the *aha1* mutant, suggesting a positive correlation between susceptibility and toxic product accumulation. Therefore, modulation of the responses by ergosterol biosynthesis genes to azoles is a mechanism by which these co-chaperones contribute to the basal azole resistance. These results suggest that rather than stabilizing ergosterol production, elimination of toxic 14α-methyl-3,6-diol might be the predominant function of the Hsp90 co-chaperones Sti1, Aha1, and P23 in *N. crassa*. It is interesting to test whether the growth defects caused by azoles would be mitigated by depleting 14α-methyl-3,6-diol from treated cells, for example, by deleting *erg8*, which encodes the phosphomevalonate kinase required for lanosterol biosynthesis (Tsay and Robinson, 1991).

Since neither Hsp90 nor its co-chaperones directly activate gene transcription, they regulate azole responsive genes likely via some transcription factors that are regulated by Hsp90's client proteins such as calcineurin. In *N. crassa* and *F. verticillioides*, transcription factors CCG-8, ADS-4, and CSP-1 regulate transcriptional responses by genes involved in ergosterol biosynthesis (Sun et al., 2014; Wang et al., 2015; Chen et al., 2016). It remains unclear whether Hsp90 dependent regulation of azole responses links to these transcription factors.

Reducing or eliminating Hsp90 functions can abolish resistance to diverse antifungals, and Hsp90 inhibition was proposed as a new way to treat fungal infections (Cowen, 2013). Although fungal Hsp90s share a high degree of conservation with human Hsp90s, fungal Hsp90 could be antifungal targets due to differential functions among Hsp90 isoforms in human and fungi cells (Chen et al., 2006; Wang et al., 2009; Cowen, 2013). Our results suggested that inhibition of Hsp90 co-chaperone Sti1, Aha1, and P23 could increase the efficacy of antifungal azoles. Thus, these proteins could be used as potential targets to develop new drugs to promote the efficacy of azoles. Combination of P23 inhibitors with other antifungal agents, such as azoles and echinocandins, could assist treatment of IFDs, especially in life-threating cases. Since homologs of each of these Hsp90 co-chaperones are highly conserved in fungi and the divergence of homologs of each co-chaperone exist between fungi and humans, it is possible to develop new drugs specifically targeting fungal Hsp90s co-chaperones. Fungal P23 might be especially suitable to be an antifungal target because human P23 lacks a disordered C-terminal domain compared with fungal homologs. Chemicals that disrupt P23 functions were previously reported. One is a nature product celastrol, which inhibits the chaperoning of steroid receptors by Hsp90 by inducing the fibrillation of P23 (Chadli et al., 2010). Another is gedunin which inactivate P23 and could cause apoptosis of cancer cells (Patwardhan et al., 2013). Therefore, it is possible to obtain fungal specific P23 inhibitors either from nature or by chemical synthesis.

## Author contributions

XS and SL contributed to the design of the work; XG, WX, and YY contributed to perform the experiments and analysis of data; XG and XS wrote the paper; SL and HL revised critically the manuscript. All authors approved the final version of the work.

## Funding

This project is supported by Grant 31370024 (to XS) from the National Natural Science Foundation of China.

### Conflict of interest statement

The authors declare that the research was conducted in the absence of any commercial or financial relationships that could be construed as a potential conflict of interest.
