# Supplementary material for: The Hsp90 Co-chaperones Sti1, Aha1, and P23 Regulate Adaptive Responses to Antifungal Azoles
Source: Front Microbiol. 2016 Oct 5;7:1571. doi: 10.3389/fmicb.2016.01571 (PMC5050212; doi:10.3389/fmicb.2016.01571)
Supplement: Supplementary file 1 [file Table1.DOCX]

**Supplementary data**

**Table S1** List of gene-specific primer pairs

| **Gene** | | **Forward Primer 5’→ 3’** | **Reversed Primer 5’→ 3’** |
| --- | --- | --- | --- |
| **QPCR primers** | | | |
| N-β-tubulin |  | CCCAAGAACATGATGGCTGCTTCT | TTGTTCTGAACGTTGCGCATCTGG |
| NCU05591 | *cdr4* | ACGCTTTGGAAATGGATGGTGACG | ATGAACAAGGCGACGGAAATGCAG |
| NCU05278 | *erg5* | TTTCACCTTCCTCTTCGCTTCCCA | TCATCGACTCAAGCTGCTCCATGT |
| NCU02624 | *erg11* | AAATCGATTACGGCTACGGTCTCG | TATCGCTACCATCCACGTTCCTGA |
| NCU08899 | *ads-1* | AACTCTGCAGTGCCAAACCTCAAC | ACAGCTGCGAGACGACATACATGA |
| NCU01898 | *ads-2* | AAGACTGTCTGGTTCGAGCCCTTT | CTCCGTTAACGATGGCAAAGCACA |
| NCU08744 | *ads-4* | TCGAACTCTTGGGACTGCCAGAAA | AAGGCATTCCGATTGAGTCCGCTA |
| NCU09686 | *ccg-8* | AAGGTGGCTCTCTCCTTTA | GGTCATTTGGTTCATCTTCTTG |
| NCU03006 | *erg6* | TCAGCTCAAGTTCGTCAAGGGTGA | TTCATAGACACCAAAGGTACCGCC |
| NCU04142 | *hsp80* | GCTTCTCTCCCTCATCATCAAC | AGGGACTCATAGCGGATCTT |
| NCU06207 | *erg3* | CTTTCTCGTCCTGGGTGTATG | TGACGGTAGATGTTGTCTCTTG |
| NCU01792 | *p23* | TTCCCGACGTCCCTACTT | CTCGACATGGTAGGTCTTCTTG |
| NCU04087 | *aha1* | ACTTCCTCCTCCTCCTCATC | GGTCAGTGAATGTAGCGTAGAG |
| NCU00714 | *sti1* | CTACTTTGAGAAGGGCGACTAC | TGACCTTGAAGTCGGCATAAC |
| NCU08512 | *hsf1* | TAAACCCAAGAGTGGAGGAAAG | GAGTAGTTCTGACCAGCGTATG |
| **Complementation** | | | |
| NCU01792 | *p23* | Nc-p23-com-1(ClaI)  CCATCGATGGAGCGGAGTAGGGTGTTGCATTT | Nc-p23-com-2(EcoRV)  ATAGATATCCCTGGCGGTAAAGTGTTCG |
| NCU00714 | *sti1* | Nc-sti1-com-1(XbaI)  GCTCTAGACAAGTAGCAGGTCCGTAAGTCGT | Nc-sti1-com-2(BamHI)  CGGGATCCCTTTTACGACCCCTTCACCTC |
| NCU04087 | *aha1* | Nc-aha1-com-1  CCATCGATACACCACGCTTGAAGACCC | Nc-aha1-com-2  CGGGATCCCTTCCTAGCTAACGCCTTTC |
| ***N. crassa* transformants and double mutant screening** | | | |
| NCU01792 | *p23* | TCATCTACCTCACCATTAGCGTTCC | CGACAGTCTCCTTGGCATCCTTT |
| NCU00714 | *sti1* | TCCTTGGTGTCCTGATGGGTGTA | ATGTGCTCGGCCAAAGCGGCAGGGT |
| NCU04087 | *aha1* | AAAACTCGGTTTCGCATGTAGGC | GGGAGATACCATGTTAGTGCGTTG |
| **Construction of *F. verticillioides* knockout strains** | | | |
|  | *p23-5’* | Fv-KO-p23-1 CTTGGAGGATAAGGTTCTTGGTC | |
|  |  | Fv-KO-p23-2 CCTGCAGCCCGGGGGATCCTGTGAAGGTTGAAGTAAAAG | |
|  | *hph* | Fv-KO-p23-3 AGGATCCCCCGGGCTGCAGGAATTC | |
|  |  | Fv-KO-p23-4 ATGATTGAGATATGGATCCCGGTCGGCATCTACTCTATTC | |
|  | *p23-3’* | Fv-KO-p23-5 TCCATATCTCAATCATGAATGTTTGACGTT | |
|  |  | Fv-KO-p23-6 CCAACCTAAGGCCCAACCC | |
|  | *sti1-5’* | Fv-KO-sti1-1 TGGGACGACCTCCTACTTGTTAC | |
|  |  | Fv-KO-sti1-2 CAGCCCGGGGGATCCTTCGTGTGAGAATTAGCAGA | |
|  | *hph* | Fv-KO-sti1-3 CACGAAGGATCCCCCGGGCTGCAGG | |
|  |  | Fv-KO-sti1-4 TCGCTTCGCGGATCCCGGTCGGCAT | |
|  | *sti1-3’* | Fv-KO-sti1-5 ACCGGGATCCGCGAAGCGAACCAGATGATG | |
|  |  | Fv-KO-sti1-6 TTCGGTTCGTCAGTTATCATAATCC | |
| ***F. verticillioides* transformants screening** | | | |
|  | *p23* | Fv-p23-check-1  TAGAACATATGAAAGCATGAAATTG | Fv-p23-check-2  TAGGTAGCGCCGACTTTCCAGGGTA |
|  | *sti1* | Fv-sti1-check-1  GATGAACTACCTCGGCGACTGGG | Fv-sti1-check-2  ACACCCTTGTAAGTGACAGACCC |
