# Supplementary material for: The Hsp90 Co-chaperones Sti1, Aha1, and P23 Regulate Adaptive Responses to Antifungal Azoles
Source: Front Microbiol. 2016 Oct 5;7:1571. doi: 10.3389/fmicb.2016.01571 (PMC5050212; doi:10.3389/fmicb.2016.01571)
Supplement: Supplementary file 2 [file Image1.PDF]

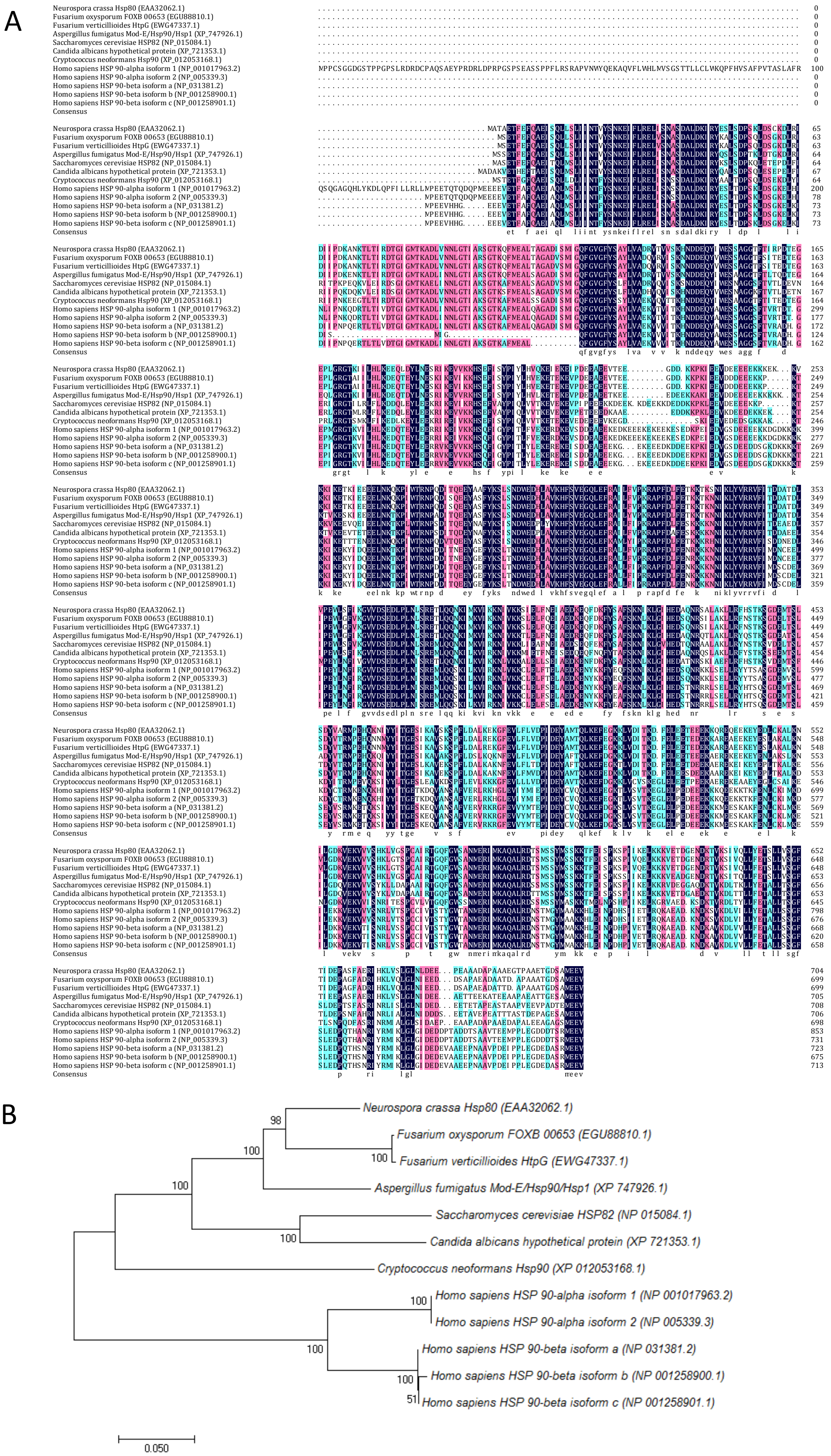
A
Neurospora crassa Hsp80 (EAA32062.1)
Fusarium oxysporum FOXB 00653 (EGU88810.1)
Fusarium verticillioides HtpG (EWG47337.1)
Aspergillus fumigatus Mod-E/Hsp90/Hsp1 (XP_747926.1)
Saccharomyces cerevisiae HSP82 (NP_015084.1)
Candida albicans hypothetical protein (XP_721353.1)
Cryptococcus neoformans Hsp90 (XP_012053168.1)
Homo sapiens HSP 90-alpha isoform 1 (NP_001017963.2)
Homo sapiens HSP 90-alpha isoform 2 (NP_005339.3)
Homo sapiens HSP 90-beta isoform a (NP_031381.2)
Homo sapiens HSP 90-beta isoform b (NP_001258900.1)
Homo sapiens HSP 90-beta isoform c (NP_001258901.1)
Consensus
B
98
100
100
100
100
100
100
100
51
Neurospora crassa Hsp80 (EAA32062.1)
Fusarium oxysporum FOXB 00653 (EGU88810.1)
Fusarium verticillioides HtpG (EWG47337.1)
Aspergillus fumigatus Mod-E/Hsp90/Hsp1 (XP 747926.1)
Saccharomyces cerevisiae HSP82 (NP 015084.1)
Candida albicans hypothetical protein (XP 721353.1)
Cryptococcus neoformans Hsp90 (XP 012053168.1)
Homo sapiens HSP 90-alpha isoform 1 (NP 001017963.2)
Homo sapiens HSP 90-alpha isoform 2 (NP 005339.3)
Homo sapiens HSP 90-beta isoform a (NP 031381.2)
Homo sapiens HSP 90-beta isoform b (NP 001258900.1)
Homo sapiens HSP 90-beta isoform c (NP 001258901.1)
0.050

Figure S1. Multiple sequence alignment and phylogenic analysis of fungal and human Hsp90 homologues. **(A)** The peptide sequences were multiple aligned with DNAman software and then **(B)** the phylogenic tree was created with Neighbour-Joining method using MEGA 7.0 software. Bootstrap support for internal branches was estimated from 1000 pseudoreplicates for the analysis.
