# Supplementary material for: The Hsp90 Co-chaperones Sti1, Aha1, and P23 Regulate Adaptive Responses to Antifungal Azoles
Source: Front Microbiol. 2016 Oct 5;7:1571. doi: 10.3389/fmicb.2016.01571 (PMC5050212; doi:10.3389/fmicb.2016.01571)
Supplement: Supplementary file 3 [file Image2.PDF]

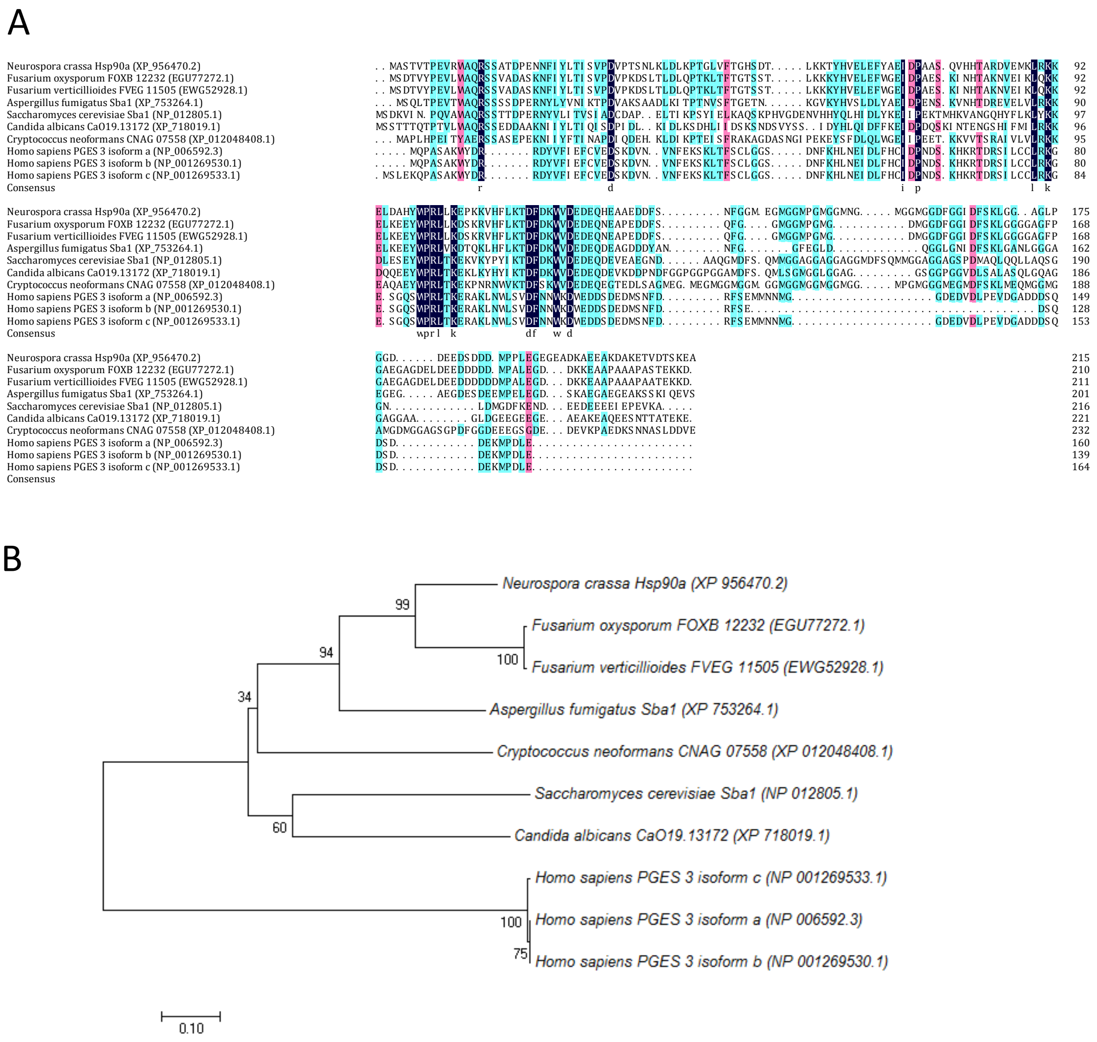

Figure S2. Multiple sequence alignment and phylogenic analysis of fungal and human P23 homologues. **(A)** The peptide sequences were multiple aligned with DNAman software and then **(B)** the phylogenic tree was created with Neighbour-Joining method using MEGA 7.0 software. Bootstrap support for internal branches was estimated from 1000 pseudoreplicates for the analysis.
